# Trajectory of efficacy and safety across ulotaront dose levels in schizophrenia: a systematic review and dose–response meta-analysis

**DOI:** 10.1093/ijnp/pyaf059

**Published:** 2025-08-08

**Authors:** Yu-Chia Hsu, Tzu-Yen Hung, Yang-Chieh Brian Chen, Kuo-Chuan Hung, Chih-Sung Liang, Ping-Tao Tseng, Yu-Kang Tu, Christoph U Correll, Chih-Wei Hsu, Marco Solmi

**Affiliations:** Department of Medical Education, National Cheng Kung University Hospital and College of Medicine, National Cheng Kung University, Tainan, Taiwan; Department of Medical Education, National Cheng Kung University Hospital and College of Medicine, National Cheng Kung University, Tainan, Taiwan; Department of Psychiatry and Behavioral Sciences, The University of Texas Health Science Center at Houston, Houston, TX, United States; Department of Anesthesiology, Chi Mei Medical Center, Tainan, Taiwan; Department of Psychiatry, Beitou Branch, Tri-Service General Hospital, National Defense Medical University, Taipei, Taiwan; Department of Psychiatry, National Defense Medical University, Taipei, Taiwan; Prospect Clinic for Otorhinolaryngology & Neurology, Kaohsiung, Taiwan; Institute of Biomedical Sciences, National Sun Yat-sen University, Kaohsiung, Taiwan; Department of Psychology, College of Medical and Health Science, Asia University, Taichung, Taiwan; Institute of Precision Medicine, National Sun Yat-sen University, Kaohsiung City, Taiwan; Institute of Health Data Analytics & Statistics, College of Public Health, National Taiwan University, Taipei, Taiwan; Health Data Research Center, National Taiwan University, Taipei, Taiwan; Department of Child and Adolescent Psychiatry, Charité Universitätsmedizin, Berlin, Germany; Department of Psychiatry, Zucker Hillside Hospital, Northwell Health, Glen Oaks, United States; Department of Psychiatry and Molecular Medicine, Zucker School of Medicine at Hofstra/Northwell, Hempstead, United States; Department of Psychiatry, Kaohsiung Chang Gung Memorial Hospital and Chang Gung University College of Medicine, Kaohsiung, Taiwan; Department of Child and Adolescent Psychiatry, Charité Universitätsmedizin, Berlin, Germany; Department of Psychiatry, University of Ottawa, Ottawa, Canada; Department of Mental Health, The Ottawa Hospital, Ottawa, Canada; Ottawa Hospital Research Institute, Ottawa, Canada; School of Epidemiology and Public Health, Faculty of Medicine, University of Ottawa, Ottawa, Canada

**Keywords:** acceptability, effectiveness, psychosis, SEP-363856, tolerance

## Abstract

**Background:**

Ulotaront is an experimental antipsychotic for schizophrenia, but its optimal dose is unclear. This study aimed to evaluate dose–response relationships for efficacy and safety in people with schizophrenia.

**Methods:**

A systematic review of four databases (until January 22, 2025; INPLASY202510091) identified randomized clinical trials assessing ulotaront. Outcomes included efficacy, measured by changes in the Positive and Negative Syndrome Scale (PANSS) total score (primary outcome), positive and negative subdomains, and the Clinical Global Impression Scale-Severity, and safety, assessed by all-cause dropout (co-primary outcome, dropout due to adverse event, serious, non-serious, and specific adverse events). We employed one-stage dose–response meta-analysis (random-effects model) calculating standardized mean differences (SMDs) and risk ratios (RRs) with 95% confidence intervals (CIs).

**Results:**

Analysis of three randomized clinical trials (*n* = 1144) indicated that the 100 mg dose of ulotaront provided the greatest improvement in PANSS total score (standardized mean difference = −0.23 [95% CI: −0.43, −0.02]), PANSS positive symptom score (−0.30 [−0.70, 0.10]), and PANSS negative symptom score (−0.28 [−0.48, −0.08]). However, Clinical Global Impression Scale-Severity scores did not exhibit a clear dose–response relationship. Regarding safety, all-cause dropout (RR at 100 mg = 1.10 [95% CI: 0.57, 2.12]), adverse event-related dropout, serious, non-serious, and most specific adverse events showed no significant dose–response relationship. The risk of anxiety-related adverse events was significantly higher than placebo at 50 and 75 mg doses (RR at 75 mg = 2.06 [95% CI: 1.11, 3.80]).

**Conclusion:**

Ulotaront 100 mg appears greatest efficacy with favorable safety for acute schizophrenia. However, effect sizes were small, and higher ulotaront doses should be tested.

**Significance Statement**

Ulotaront is a new medication being tested for treating schizophrenia. Unlike most existing antipsychotic drugs that block dopamine receptors in the brain, ulotaront works through a different mechanism by activating trace amine-associated receptor 1 and serotonin 1A receptors. These novel targets may help reduce both hallucinations and negative symptoms like social withdrawal and lack of motivation, with fewer side effects. In this study, we analyzed data from several clinical trials to understand how different doses of ulotaront affect patients. We found that higher doses—especially around 100 mg—can improve schizophrenia symptoms without increasing safety concerns. These findings are important because they suggest that ulotaront may offer a new and safer treatment option for people with schizophrenia, and they help guide doctors toward the most effective dose.

## INTRODUCTION

Schizophrenia is a chronic and severe psychiatric condition with a global prevalence of ~1%.^1^ Schizophrenia is characterized by a combination of positive symptoms (hallucinations, delusions, and disorganized thoughts and behavior) and negative symptoms (avolition, anhedonia, affective flattening, alogia, and social withdrawal) as well as cognitive impairment.^1^ Without continued antipsychotic treatment, schizophrenia can severely impact patients' ability to function and is among the top 10 global causes of disability.^2^ Current antipsychotic medications primarily target dopamine receptors, particularly the postsynaptic D2 receptor.^3^ While ~30% of patients experience substantial symptom improvement, a considerable proportion continue to experience persistent psychotic or residual symptoms owing to treatment resistance—22% in first-episode and up to 39.5% in multiple-episode cohorts—thereby compromising their quality of life.^4^^,^^5^ Research indicates that antipsychotics are more effective in reducing positive symptoms; however, up to 40% of patients do not respond to treatment for positive symptoms and are considered treatment-resistant to current dopamine receptor antagonists.^4^ The challenge is even greater for negative symptoms and cognitive dysfunction, as up to 60% of patients continue to experience predominant negative symptoms despite treatment,^6^^,^^7^ and almost all patients demonstrate cognitive dysfunction compared to healthy controls or their predicted cognitive functioning based on maternal factors.^8^ Furthermore, while antipsychotics can alleviate symptoms, their therapeutic benefits are often undermined by intolerable adverse effects,^9^^,^^10^ resulting in poor patient adherence and treatment discontinuation.^11^ These challenges underscore the urgent need for novel antipsychotic medications that can effectively address both positive and negative symptoms as well as cognitive functioning, while minimizing the adverse effects.^12^

To date, several novel compounds have been developed for the treatment of schizophrenia,^13-15^ and ulotaront has emerged as a potential candidate.^16^ Ulotaront acts as a trace amine-associated receptor 1 (TAAR1) and serotonin 1A (5-HT1A) receptor agonist, with high affinity for TAAR1.^17^ Unlike traditional antipsychotics, ulotaront has exhibited antipsychotic properties without directly binding to the D2 receptor.^18^ Previously, a phase 2 randomized controlled trial (RCT) showed superior efficacy versus placebo in improving both positive and negative symptoms.^19^ However, the subsequent phase 3 DIAMOND 1 and 2 RCTs yielded disappointing results due to a high placebo effect,^20^ raising concerns about ulotaront’s consistent efficacy. Given these inconsistent findings, a comprehensive investigation is required to determine whether treatment response variates across different doses. Recently, a systematic review by Siafis et al. analyzed the efficacy of ulotaront and supported its effectiveness against psychotic symptoms.^21^ However, that review did not separately evaluate positive and negative symptoms, leaving unanswered questions about whether overall improvements are mirrored in the subdomains. Furthermore, the relationship between dose and treatment response remains unclear. The phase 2 trial used an average dose of ~70 mg and demonstrated favorable results for total PANSS.^19^ In contrast, the DIAMOND 1 trial found that the 50 mg dose was less effective than placebo and the 75 mg dose showed slight numerical improvement,^20^ while in DIAMOND 2, both the 75 and the 100 mg doses of ulotaront showed slight numerical improvements over placebo.^20^ These findings raise the question of whether higher doses of ulotaront could result in greater efficacy, warranting further systematic quantitative investigation.

A dose–response meta-analysis can be a valuable tool to tackle this issue. This method analyzes the relationship between a drug's dosage and its therapeutic outcomes.^22^^,^^23^ By using this approach, clinicians can gain insights into the optimal dose or dose range that maximizes therapeutic benefits. This methodology has been applied in prior research on antipsychotic medications. For example, a dose–response meta-analysis of lurasidone for bipolar depression found that doses between 40 and 60 mg provided better outcomes than higher doses, offering a balance between effectiveness and safety.^24^ To bridge the knowledge gap on the role of ulotaront in the treatment of schizophrenia-spectrum disorders, this study attempted to systematically include all available placebo-controlled RCTs with ulotaront in schizophrenia and employed a dose–response meta-analysis. The analyses aimed to evaluate ulotaront’s efficacy in treating psychotic symptoms, particularly positive and negative symptoms, while also examining its safety profile by analyzing dropout rates and adverse events.

## METHODS

### Search Strategy and Study Selection

The study was registered with the International Platform of Registered Systematic Review and Meta-Analysis Protocols (INPLASY202510091) and followed the guidelines outlined in the Preferred Reporting Items for Systematic Reviews and Meta-Analyses 2020 statement (Supplementary Table S1).^25^

A comprehensive literature search was conducted across the following databases: PubMed, Embase, Cochrane Library, and ClinicalTrials.gov. The search used the keywords (SEP-363856 OR SEP-856 OR ulotaront) AND (psychosis OR psychotic disorder OR schizophreni* OR schizoaffective disorder OR delusional disorder) and included all relevant literature published up to January 22, 2025, without restrictions on language or geographic region. Detailed search strings are provided in Supplementary Table S2. The PICOS framework was structured as follows: Patient: adults diagnosed with schizophrenia-spectrum disorder; Intervention: treatment with ulotaront; Comparison: placebo as the comparator; Outcome: the primary outcome of interest was the change in total psychopathology; and Study design: RCTs.

The primary aim of this systematic review and meta-analysis was to evaluate the dose–response relationship of ulotaront in the treatment of schizophrenia-spectrum disorders during the acute treatment phase. Studies meeting the following criteria were included: (1) participants had a diagnosis of a schizophrenia-spectrum disorder based on established diagnostic criteria, such as the Diagnostic and Statistical Manual of Mental Disorders or the International Classification of Diseases; (2) reported quantitative data on clinical outcomes, including severity of total psychopathology measured with a validated scale (e.g., the Positive and Negative Syndrome Scale [PANSS]) before and after medication administration in the acute phase. The following exclusion criteria were applied: (1) non-RCTs or studies using comparators other than placebo—as this review assumed that the placebo serves as a zero-dose baseline for ulotaront, trials comparing ulotaront with other drugs or those lacking a placebo arm were excluded, since they do not provide relevant data for dose–response analyses; (2) studies involving participants without a confirmed diagnosis of a schizophrenia-spectrum disorder based on an established criteria; (3) studies that did not report quantitative clinical outcomes appropriate for dose–response analysis; and (4) duplicate data from research protocols or secondary publications. In cases of multiple publications originating from the same research source, only the report with the largest sample size and the most comprehensive data was included.

Two independent reviewers (Y.C.H. and C.W.H.) screened titles, abstracts, and full texts. In cases of disagreement, discussions were held between the two reviewers to reach a consensus. Where necessary, a third-party adjudicator (T.Y.H.) was consulted to resolve any disputes.

### Data Extraction and Quality Assessment

Data extraction was performed independently by two reviewers (Y.C.H. and C.W.H.). For each included study, information was collected on study design, participant demographics (e.g., age, sex, sample size, and baseline disease severity), and intervention details (e.g., dosage and treatment duration). Outcome data were classified into two primary domains: treatment efficacy and safety. For treatment efficacy, the primary outcome was the change in the severity of total psychopathology for the placebo and treatment groups, assessed by the PANSS total scores. Secondary outcomes included the PANSS positive symptom subscore, PANSS negative symptom subscore, and the Clinical Global Impression Scale-Severity (CGI-S). For treatment safety, the primary outcome was all-cause dropout during the study period. Secondary outcomes included dropout due to adverse effect and adverse effect rates. Adverse effects were categorized into serious and non-serious events as per the authors’ definition. Additionally, we examined specific adverse events mentioned in the phase 2 study of ulotaront,^19^ such as extrapyramidal symptoms, insomnia, somnolence, headache, nausea, agitation, worsening schizophrenia symptoms, and anxiety.

The risk of bias for each included study was assessed using the Cochrane Risk of Bias Tool, Version 2 (RoB 2).^26^ This evaluation was conducted independently by two reviewers (Y.C.H. and C.W.H.), with any disagreements resolved through discussion to ensure an accurate and consensus-based assessment of study quality.

### Data Synthesis and Statistical Analysis

For PANSS and CGI-S, pre- to post-treatment changes were calculated and expressed as standardized mean differences (SMDs) with 95% confidence intervals (CIs). For dropout and adverse events, event counts were converted to risk ratios (RRs) with 95% CIs. To investigate whether ulotaront exhibited a dose-dependent relationship compared with placebo for both primary and secondary outcomes, a one-stage random-effects dose–response meta-analysis was conducted.^27^ Dose–response curves were modeled using restricted cubic splines with three knots placed at fixed percentiles (10%, 50%, and 90%).^28^ Model fit was assessed using goodness-of-fit statistics, with the coefficient of determination (*R*^2^) reflecting the proportion of effect-size variability explained by dose.^29^ Heterogeneity in the one-stage dose–response meta-analysis was evaluated using the variance partition coefficient, an extension of the *I*^2^ statistic.^30^ All statistical analyses were performed in R version 4.3.2 (R Project for Statistical Computing). Two-sided tests were used with alpha <0.05.

For the primary efficacy outcome, two additional analyses were conducted. First, a leave-one-out analysis was performed to assess the impact of excluding individual studies on the overall findings. Second, we assessed the time-course of treatment efficacy by comparing ulotaront with placebo. We also stratified ulotaront treatment groups by dose to investigate potential efficacy. This analysis followed the same methodology as the primary dose–response analysis and was repeated accordingly.

## RESULTS

The systematic search across multiple databases and registers identified a total of 283 records (Supplementary Figure S1). After removing 96 duplicates, 187 unique records were screened. Of these, 177 records were excluded based on title and abstract screening. This left 10 reports for retrieval, all of which were successfully retrieved and assessed for eligibility. Following a full-text review, 7 reports were excluded for reasons detailed in Supplementary Table S3. Ultimately, three studies were included in this analysis: Koblan et al., DIAMOND 1 trial (NCT04072354), and DIAMOND 2 trial (NCT04092686).^19^^,^^20^ Collectively, these trials enrolled 1144 participants (mean age = 35.8 years; 40.1% female), the majority of whom were white (72%-82%) (Table 1). The key eligibility criteria and procedural features of the three included studies were as follows: (1) participants could have mild-to-moderate mood dysphoria or anxiety but no other major psychiatric diagnoses (e.g., major depressive disorder, bipolar disorder, obsessive-compulsive disorder, post-traumatic stress disorder, or substance-use disorder); (2) schizophrenia had to be present for ≥6 months with ≤2 prior hospitalizations; and (3) all therapeutic psychotropic agents (antipsychotics, antidepressants, mood stabilizers, regular tranquillizers) were discontinued, and only tightly restricted rescue or supportive medications were permitted, such as lorazepam for acute agitation, single-dose hypnotics at bedtime, anticholinergics for extrapyramidal symptoms, or propranolol for akathisia.

**Table 1:** Characteristics of included studies

**Study**	**Study design**	**Diagnosis**	**Age, year (SD)**	**Cases number (male/female)**	**Treatment group, dose**	**Treatment duration**	**Assessment tools**	**Region/ethnicity**
**Koblan 2020**	Double-blind, parallel	Schizophrenia (DSM-5)	30.6 (6.1) 30.0 (5.8)	125 (79/46) 120 (77/43)	Placebo Ulotaront 50 or 75 mg	4 weeks	PANSS, CGI-S, MARDS, BNSS	USA, Europe White (82%), Black (16%), Other (2%)
**DIAMOND 1** ^**a**^ **(NCT04072354)**	Double-blind, parallel	Schizophrenia (DSM-5)	35.7 (10.3) 36.1 (9.4) 37.0 (10.2)	146 (73/73) 144 (98/46) 145 (86/59)	Placebo Ulotaront 50 mg Ulotaront 75 mg	6 weeks	PANSS, CGI-S, BNSS	USA, Europe White (78%), Black (21%), Other (1%)
**DIAMOND 2** ^**a**^ **(NCT04092686)**	Double-blind, parallel	Schizophrenia (DSM-5)	38.6 (10.8) 38.5 (11.2) 37.5 (10.3)	155 (90/65) 155 (84/71) 154 (98/56)	Placebo Ulotaront 75 mg Ulotaront 100 mg	6 weeks	PANSS, CGI-S, BNSS	USA, Europe White (72%), Black (24%), Other (4%)

Abbreviations: BNSS, Brief Negative Symptom Scale; CGI-S, Clinical Global Impression-Severity; DSM-5, Diagnostic and Statistical Manual of Mental Disorders, fifth edition; MADRS, Montgomery-Asberg Depression Rating Scale; PANSS, Positive and Negative Syndrome Scale.

a A poster presented at the American College of Neuropsychopharmacology provides data from the DIAMOND 1 and DIAMOND 2 studies. The poster can be accessed online via the following link: https://cnsscientificposters.ipostersessions.com/Default.aspx?s=30-4A-E4-FB-51-B0-CE-51-94-ED-E5-96-B7-42-A4-D0

The trial by Koblan et al. utilized a two-arm design (placebo vs. flexible 50 mg or 75 mg dose) and evaluated outcomes over a 4-week treatment period.^19^ Ulotaront significantly improved PANSS total score (placebo vs. 50/75 mg: −9.7 vs. −17.2 [significant]).^19^ The DIAMOND 1 trial employed a three-arm design (placebo vs. 50 mg vs. 75 mg) and spanned 6 weeks.^20^ A relatively high placebo response was observed, with only 75 mg ulotaront providing better efficacy in improving PANSS total score (placebo vs. 50 mg vs. 75 mg: −19.3 vs. −16.9 [not significant] vs. −19.6 [not significant]).^20^ The DIAMOND 2 trial, which involved three arms (placebo vs. 75 mg vs. 100 mg) over 6 weeks, also detected a high placebo effect. Nevertheless, the trial found greater efficacy with higher doses of ulotaront in reducing the PANSS total score (placebo vs. 75 mg vs. 100 mg: −14.3 vs. −16.4 [not significant] vs. −18.1 [significant]).^20^

**Figure 1:**
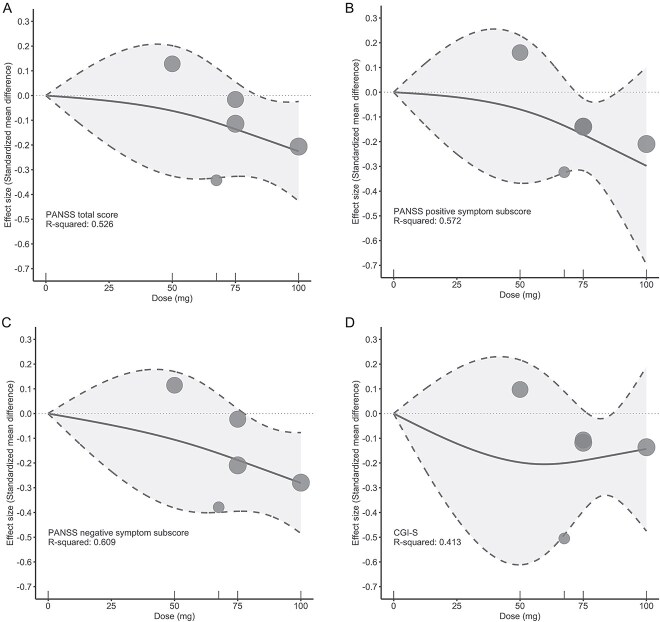
Dose–response relationship between ulotaront doses and PANSS score. (**A**) PANSS total score; (**B**) PANSS positive symptom subscore; (**C**) PANSS negative symptom subscore; and (**D**) CGI-S score. Solid line: Pooled point estimates; dotted line: 95% confidence interval; short vertical lines on the *x*-axis: Ulotaront dose of the included studies; open circles: Outcome markers for all included studies, size of which represents the reciprocal of the standard error of the effect size. CGI-S, Clinical Global Impression-Severity; PANSS, Positive and Negative Syndrome Scale.

The analysis of the PANSS total score showed that higher fixed doses of ulotaront were associated with marginally greater efficacy (Figure 1A and Table 2), with optimal and significant effects observed with the 75 mg and, especially, the 100 mg dose (75 mg: SMD, −0.14; 95% CI, −0.33 to 0.05; 100 mg: SMD, −0.23; 95% CI, −0.43 to −0.02). The leave-one-out analysis of the PANSS total score yielded similar findings, supporting the optimal dose range of 75-100 mg (Supplementary Figure S2). Furthermore, both PANSS subscores exhibited marginal dose–response relationships similar to the PANSS total score (Figure 1B and C), with the greatest effect sizes observed within the higher doses (75-100 mg range) for positive symptoms (75 mg: SMD, −0.17; 95% CI, −0.32 to −0.03; 100 mg: SMD, −0.30; 95% CI, −0.70 to 0.10) and negative symptoms (75 mg: SMD, −0.19; 95% CI, −0.39 to 0.02; 100 mg: SMD, −0.28; 95% CI, −0.48 to −0.08). However, the CGI-S score did not demonstrate a clear dose–response relationship (Figure 1D). Finally, the temporal trend in efficacy showed that in groups including 75 mg or higher doses, the absolute value of the pooled effect size gradually increased over time, whereas this trend was not observed in the dose group of only 50 mg (Supplementary Figure S3).

**Table 2:** Estimated effect sizes from dose–response meta-analysis

	**Ulotaront dose** ^**a**^			
**Outcome (*N* studies)**	**25 mg**	**50 mg**	**75 mg**	**100 mg**
**Efficacy (standardized mean difference)**				
**PANSS total score (*N* = 3)**	−0.02 (−0.20, 0.16)	−0.06 (−0.33, 0.20)	−0.14 (−0.33, 0.05)	−0.23 (−0.43, −0.02)
**PANSS positive symptom subscore (*N* = 3)**	−0.02 (−0.25, 0.21)	−0.07 (−0.37, 0.23)	−0.17 (−0.32, −0.03)	−0.30 (−0.70, 0.10)
**PANSS negative symptom subscore (*N* = 3)**	−0.05 (−0.23, 0.14)	−0.11 (−0.38, 0.17)	−0.19 (−0.39, 0.02)	−0.28 (−0.48, −0.08)
**CGI-S (*N* = 3)**	−0.12 (−0.42, 0.18)	−0.20 (−0.61, 0.22)	−0.19 (−0.39, 0.01)	−0.14 (−0.47, 0.19)
**Safety (risk ratio)**				
**All-cause dropout (*N* = 3)**	1.09 (0.84, 1.40)	1.14 (0.82, 1.59)	1.14 (0.85, 1.52)	1.10 (0.57, 2.12)
**Dropout due to adverse event (*N* = 3)**	1.31 (0.76, 2.26)	1.53 (0.76, 3.07)	1.43 (0.86, 2.37)	1.20 (0.33, 4.39)
**Severe adverse event (*N* = 3)**	1.50 (0.88, 2.55)	1.95 (0.88, 4.33)	1.95 (0.96, 3.94)	1.72 (0.72, 4.10)
**Non-severe adverse event (*N* = 3)**	1.13 (0.93, 1.37)	1.21 (0.90, 1.62)	1.17 (0.85, 1.62)	1.09 (0.66, 1.78)
**Extrapyramidal symptoms (*N* = 3)**	0.94 (0.48, 1.83)	0.94 (0.37, 2.40)	1.04 (0.50, 2.15)	1.21 (0.36, 4.00)
**Insomnia (*N* = 3)**	1.26 (0.83, 1.91)	1.23 (0.61, 2.46)	0.75 (0.24, 2.38)	0.37 (0.05, 2.84)
**Headache (*N* = 2)**	1.33 (0.92, 1.92)	1.59 (0.91, 2.77)	1.55 (0.96, 2.48)	1.32 (0.72, 2.42)
**Nausea (*N* = 3)**	1.16 (0.74, 1.84)	1.29 (0.67, 2.46)	1.29 (0.69, 2.40)	1.24 (0.41, 3.78)
**Agitation (*N* = 3)**	1.20 (0.71, 2.02)	1.29 (0.64, 2.62)	1.16 (0.66, 2.05)	0.96 (0.29, 3.15)
**Schizophrenia (*N* = 3)**	1.31 (0.56, 3.09)	1.61 (0.45, 5.72)	1.74 (0.68, 4.46)	1.79 (0.82, 3.92)
**Anxiety (*N* = 2)**	1.55 (1.01, 2.40)	2.07 (1.07, 4.03)	2.06 (1.11, 3.80)	1.69 (0.74, 3.84)

Abbreviations: CGI-S, Clinical Global Impression-Severity; PANSS, Positive and Negative Syndrome Scale.

a 0 mg ulotaront is the reference group and gray background indicates statistical significance.

**Figure 2:**
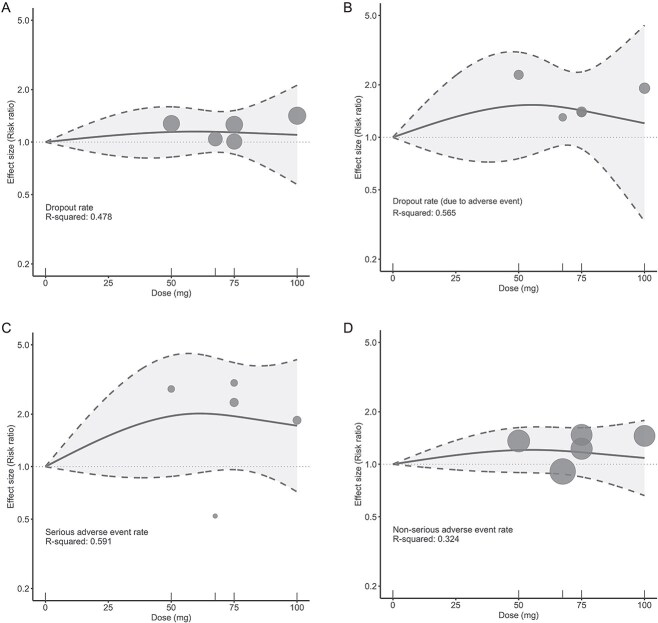
Dose–response relationship between daily ulotaront doses and safety. (**A**) All-cause dropout rate; (**B**) dropout rate due to adverse event; (**C**) serious side effect rate; and (**D**) non-serious side effect rate. Solid line: Pooled point estimates; dotted line: 95% confidence interval; short vertical lines on the *x*-axis: Ulotaront dose of the included studies; open circles: Outcome markers for all included studies, size of which represents the reciprocal of the standard error of the effect size.

In terms of all-cause dropout rate (Fig. 2a and Table 2), no specific dose of ulotaront showed a significantly higher risk compared to placebo (50 mg: RR, 1.14; 95% CI, 0.82 to 1.59; 75 mg: RR, 1.14; 95% CI, 0.85 to 1.52; 100 mg: RR, 1.10; 95% CI, 0.57 to 2.12). Other secondary safety outcomes, including dropout due to adverse event, and serious and non-serious adverse events, exhibited a similar non-relevant dose–response relationship, without significantly increased risk compared to placebo (Figure 2B–D and Table 2). For most of the subcategories of adverse events, including extrapyramidal symptoms, insomnia, headache, nausea, agitation, schizophrenia, no specific dose was identified to be associated with a significantly higher risk compared to placebo (Supplementary Figure S4A–F and Supplementary Table S2). However, for the adverse event of anxiety (Supplementary Figure S4G and Supplementary Table S2), doses lower than 100 mg were associated with a significantly higher risk compared to placebo (50 mg: RR, 2.07; 95% CI, 1.07 to 4.03; 75 mg: RR, 2.06; 95% CI, 1.11 to 3.80). Only one RCT reported the adverse event of somnolence,^19^ so a meta-analysis was not performed (placebo: 6 cases; ulotaront: 8 cases).

A detailed quality assessment of the included studies was conducted using the RoB 2 tool, as shown in Supplementary Table S4 and Supplementary Figure. S5. All three studies were determined to have a low risk of bias across all evaluated domains. The variance partition coefficients for the PANSS total score and dropout rate are presented in Supplementary Figure S6A and B.

## DISCUSSION

The findings of this meta-analysis suggest that higher doses of ulotaront (75-100 mg) demonstrated superior efficacy versus placebo in some clinical outcomes, albeit with small effect sizes, including statistically significant improvements in the PANSS total score (100 mg), as well as the positive (75 mg) and negative symptom (100 mg) subscores. Additionally, ulotaront exhibited a favorable safety profile across all dose levels for most safety outcomes. Specifically, the dose–response analysis revealed no evidence that any dose of ulotaront was associated with an increased risk of all-cause dropout, dropout due to adverse events, serious adverse events, or non-serious adverse events. Among specific adverse events, anxiety was the only event linked to a significantly higher risk at lower doses (50-75 mg). Other adverse events, such as extrapyramidal symptoms, insomnia, headache, nausea, agitation, and worsening of schizophrenia, did not exhibit any clear dose–response relationship.

Ulotaront, a novel antipsychotic under investigation for schizophrenia and adjunctive treatment for major depressive disorder^31^ and generalized anxiety disorder,^32^ primarily exerts its antipsychotic efficacy as a TAAR1 agonist but also has 5HT1A agonist activity, which may improve depression and anxiety.^33^ Previous studies suggest that ulotaront’s efficacy in treating schizophrenia is linked to its modulation of monoaminergic circuits, particularly in regulating dopaminergic activity.^34^  *In vivo* studies have shown that TAAR1 agonists suppress dopaminergic neuronal firing in the ventral tegmental area,^33^^,^^34^ while human neuroimaging studies support their downstream regulation of striatal dopamine synthesis and release.^35^^,^^36^ Whether this mechanism follows a positive dose–response relationship remains unclear; however, our study provides evidence supporting the potential role of the TAAR1 agonist ulotaront, especially at the already tested 100 mg dose in treating schizophrenia. Specifically, efficacy appears absent to limited at lower doses but becomes apparent as the dose increases, achieving statistical significance at ~80 mg. This finding aligns with some phase 1 pharmacokinetic studies of ulotaront, which demonstrated linear dose proportionality across the 0-100 mg range.^37^ These results suggest a clinically relevant correlation between ulotaront’s dosage, plasma concentration, and clinical efficacy within this range, with no observed plateau phase. Based on this and the exceptional safety of ulotaront, demonstrated by the absence of dose–response relationship with most adverse events, doses higher than 100 mg should be explored aiming to maximize its efficacy. This exploration of higher doses is particularly needed, as the observed effect sizes in our analyses for the higher ulotaront doses were small. Furthermore, our leave-one-out analysis confirmed that excluding any individual trial did not alter the trend of higher doses yielding better efficacy.

The overall effect size of ulotaront at the 100 mg dose was small (approximately SMD = 0.2-0.3), and several factors may account for this observation. This meta-analysis incorporated three RCTs: Koblan et al. reported a more robust therapeutic effect,^19^ whereas DIAMOND 1 and DIAMOND 2 did not demonstrate relevant efficacy,^20^ resulting in a pooled effect size that fell between these divergent findings. Moreover, the relatively weak outcomes observed in DIAMOND 1 and DIAMOND 2 were predominantly driven by a higher placebo response,^20^ potentially linked to the fact that both studies were conducted during the COVID-19 pandemic. Evidence suggests that trials carried out during this period often show heightened placebo effects,^38^ potentially masking ulotaront’s true clinical efficacy. In the context of schizophrenia, the COVID-19 pandemic has reportedly increased social isolation, heightened stress, reduced social support, and disrupted mental health care in patients,^39^ factors that might have been partially ameliorated by hospitalization and regular in-person trial visits. Such contact could have contributed to the placebo response in these studies, thus diminishing the apparent difference between ulotaront and placebo.

In terms of safety, the primary outcome of all-cause dropout rate did not significantly increase compared to the placebo group across dose levels. Similarly, the secondary outcomes including dropout due to adverse effects, and serious and non-serious adverse effects showed no significant dose–response relationships. This finding indicates that ulotaront is generally safe within the 50-100 mg dose range. A previous long-term extension, 26-week study also demonstrated that ulotaront was a safe treatment option,^40^ consistent with the pooled findings of the acute 4-6-week trials. However, it is noteworthy that anxiety was the only adverse effect that occurred significantly more frequent with ulotaront versus placebo, with the highest risk observed in the 50-75 mg dose range (RR = 2). Interestingly, ulotaront is currently being investigated in clinical trials for generalized anxiety disorder, with 50 and 75 mg as the treatment doses.^32^ Paradoxically, these same doses correspond to the range that showed—based on results of this meta-analysis—the highest relative risk of treatment-emergent anxiety. The discrepancy likely reflects fundamental differences in the origins of anxiety in schizophrenia versus primary anxiety disorders. In schizophrenia, anxiety frequently stems from persecutory delusions or auditory hallucinations,^41^^,^^42^ depressive or guilt-laden affect,^43^ or distress linked to heightened illness insight.^44^ We therefore speculate that lower doses (50-75 mg) may accentuate insight-related anxiety in this population. Conversely, the 100 mg dose was not significantly associated with anxiety, suggesting that higher doses could yield stronger anxiolytic or antidepressant benefits with fewer anxiety-related adverse events in ongoing trials for generalized anxiety disorder^32^ and major depressive disorder.^31^ These interpretations remain provisional: anxiety events in our meta-analysis were self-reported and aggregated across the entire study period, with no sex-stratified data or event-timing information. Consequently, we could not assess potential gender differences or determine whether anxiety onset coincided with improvements in positive or negative symptoms.

The strength of this dose–response meta-analysis lies in its provision of insights into the optimal dosing range of ulotaront for treating schizophrenia. However, several limitations should be carefully considered. First, only three studies were included, limiting the overall strength of evidence. Although we conducted a leave-one-out analysis that yielded consistent results, supporting the reliability of our findings, the small number of studies remains a constraint. Second, only one group of participants received the 100 mg dose, which we hypothesize to be within the most effective range. Due to this limitation, some outcomes, such as the PANSS positive symptom subscore, demonstrated the best effect size yet failed to achieve statistical significance. Third, the limited number of studies lacked detailed demographic information to allow for subgroup analyses by age (e.g., pediatric or elderly populations) or ethnicity; these factors could plausibly influence both overall responsiveness to ulotaront and dose-specific effects. Fourth, although we reported all-cause dropouts and dropouts attributable to adverse events (Figure 2A and B), the reasons underlying other dropouts remain unspecified. Finally, while we hypothesize that doses higher than 100 mg could offer even greater efficacy without compromising safety, no studies have yet investigated ulotaront at doses exceeding 100 mg. Future RCTs exploring higher doses of ulotaront for the treatment of schizophrenia are warranted to better determine its optimal dose–response relationship and to optimize the efficacy-to-safety balance for managing patients with schizophrenia.

## CONCLUSION

This dose–response meta-analysis included 1144 participants and evaluated the efficacy and safety of ulotaront across doses between 50 and 100 mg. Higher doses (75-100 mg) of ulotaront showed increased efficacy, as reflected in improvements in PANSS total, positive symptom, and negative symptom scores, although effect sizes were small. No clear dose–response relationship was observed for most safety outcomes, including dropout events, dropout due to adverse events, serious adverse events, and non-serious adverse events. Currently, no trials have investigated doses of ulotaront exceeding 100 mg. Future studies could explore higher doses to identify potentially enhanced therapeutic effects while maintaining the observed tolerability of ulotaront at currently tested doses.

## Supplementary Material

Supplement_revise_pyaf059

## Data Availability

The data supporting the findings of this study are available from the corresponding author, C.W.H., upon reasonable request.

## Patient and Public Involvement

The current study did not directly involve individual participants; therefore, we did not have the opportunity to approach individual participants or explore their information. Therefore, it was impossible to obtain consent for participation in this study. The current study did not directly involve individual participants; therefore, we did not have the opportunity to approach individual participants or explore their information. Therefore, it was impossible to obtain consent for publication of this study.
