# Supplementary material for: Trajectory of efficacy and safety across ulotaront dose levels in schizophrenia: a systematic review and dose–response meta-analysis
Source: Int J Neuropsychopharmacol. 2025 Aug 8;28(9):pyaf059. doi: 10.1093/ijnp/pyaf059 (PMC12421877; doi:10.1093/ijnp/pyaf059)
Supplement: Supplement_revise_pyaf059 [file supplement_revise_pyaf059.docx]

**Supplemental data**

**Trajectory of Efficacy and Safety Across Ulotaront Dose Levels in Schizophrenia: A Systematic Review and Dose-Response Meta-Analysis**

**Running Title:** ulotaront for schizophrenia

Yu-Chia Hsu, MD^a,1^, Tzu-Yen Hung, MD^a^^,1^, Yang-Chieh Brian Chen, MD^b^, Kuo-Chuan Hung, MD^c^, Chih-Sung Liang, MD^d,e^, Ping-Tao Tseng, MD^f,g,h,i^, Yu-Kang Tu, PhD^j,k^, Christoph U. Correll, MD^l,m,n^, Chih-Wei Hsu, MD^o,2,*^, Marco Solmi, MD^l,p,q,r,s,2^

^a^ Department of Medical Education, National Cheng Kung University Hospital and College of Medicine, National Cheng Kung University, Tainan, Taiwan

^b^ Department of Psychiatry and Behavioral Sciences, The University of Texas Health Science Center at Houston, Houston, TX, USA

^c^ Department of Anesthesiology, Chi Mei Medical Center, Tainan, Taiwan

^d^ Department of Psychiatry, Beitou Branch, Tri-Service General Hospital, National Defense Medical University, Taipei, Taiwan

^e^ Department of Psychiatry, National Defense Medical University, Taipei, Taiwan

^f^ Prospect Clinic for Otorhinolaryngology & Neurology, Kaohsiung, Taiwan

^g^ Institute of Biomedical Sciences, National Sun Yat-sen University, Kaohsiung, Taiwan

^h^ Department of Psychology, College of Medical and Health Science, Asia University, Taichung, Taiwan

^i^ Institute of Precision Medicine, National Sun Yat-sen University, Kaohsiung City, Taiwan

^j^ Institute of Health Data Analytics & Statistics, College of Public Health, National Taiwan University, Taipei, Taiwan

^k^ Health Data Research Center, National Taiwan University, Taipei, Taiwan

^l^ Department of Child and Adolescent Psychiatry, Charité Universitätsmedizin, Berlin, Germany

^m^ Department of Psychiatry, Zucker Hillside Hospital, Northwell Health, Glen Oaks, USA

^n^ Department of Psychiatry and Molecular Medicine, Zucker School of Medicine at Hofstra/Northwell, Hempstead, USA

^o^ Department of Psychiatry, Kaohsiung Chang Gung Memorial Hospital and Chang Gung University College of Medicine, Kaohsiung, Taiwan

^p^ Department of Psychiatry, University of Ottawa, Ottawa, Canada

^q^ Department of Mental Health, The Ottawa Hospital, Ottawa, Canada

^r^ Ottawa Hospital Research Institute, Ottawa, Canada

^s^ School of Epidemiology and Public Health, Faculty of Medicine, University of Ottawa, Ottawa, Canada

^1^ contributed equally as first authors

^2^ contributed equally as last authors

*** Corresponding author:**

Chih-Wei Hsu

Department of Psychiatry, Kaohsiung Chang Gung Memorial Hospital

No. 123, Dapi Road, Niaosong District, Kaohsiung City 833, Taiwan

Telephone number: 886-7-7317123 ext. 8753

E-mail address: [harwicacademia@gmail.com](mailto:harwicacademia@gmail.com)

| **Content** | **Page** |
| --- | --- |
| eTable 1. PRISMA Checklist | 1-2 |
| eTable 2. Detailed search strategy | 3 |
| eTable 3. Excluded studies and reasons | 4 |
| eTable 4. Detailed quality assessment of included studies using Cochrane risk of bias 2 tool | 5 |
| eFigure 1. Flowchart of study selection | 6 |
| eFigure 2. Leave-one-out analysis of the dose-response relationship between ulotaront dose and PANSS total score | 7 |
| eFigure 3. Time-effect relationship of PANSS total score | 8 |
| eFigure 4. Dose-response relationships between ulotaront doses and specific adverse events | 9-15 |
| eFigure 5. Summary of quality assessment of included studies using Cochrane risk of bias 2 tool | 16 |
| eFigure 6. Variation partition coefficients of primary outcomes | 17-18 |
| References | 19 |

**Supplementary Table 1. PRISMA Checklist**

| **Section and Topic** | **#** | **Checklist item** | **Location** |
| --- | --- | --- | --- |
| **TITLE** | | |  |
| Title | 1 | Identify the report as a systematic review. | 1 |
| **ABSTRACT** | | |  |
| Abstract | 2 | See the PRISMA 2020 for Abstracts checklist. | 4 |
| **INTRODUCTION** | | |  |
| Rationale | 3 | Describe the rationale for the review in the context of existing knowledge. | 5-7 |
| Objectives | 4 | Provide an explicit statement of the objective(s) or question(s) the review addresses. | 5-7 |
| **METHODS** | | |  |
| Eligibility criteria | 5 | Specify the inclusion and exclusion criteria for the review and how studies were grouped for the syntheses. | 8-9 |
| Information sources | 6 | Specify all databases, registers, websites, organisations, reference lists and other sources searched or consulted to identify studies. Specify the date when each source was last searched or consulted. | 8 |
| Search strategy | 7 | Present the full search strategies for all databases, registers and websites, including any filters and limits used. | Supplementary Table 2 |
| Selection process | 8 | Specify the methods used to decide whether a study met the inclusion criteria of the review, including how many reviewers screened each record and each report retrieved, whether they worked independently, and if applicable, details of automation tools used in the process. | 9 |
| Data collection process | 9 | Specify the methods used to collect data from reports, including how many reviewers collected data from each report, whether they worked independently, any processes for obtaining or confirming data from study investigators, and if applicable, details of automation tools used in the process. | 9-10 |
| Data items | 10a | List and define all outcomes for which data were sought. Specify whether all results that were compatible with each outcome domain in each study were sought (e.g., for all measures, time points, analyses), and if not, the methods used to decide which results to collect. | 9 |
|  | 10b | List and define all other variables for which data were sought (e.g., participant and intervention characteristics, funding sources). Describe any assumptions made about any missing or unclear information. | 9 |
| Study risk of bias assessment | 11 | Specify the methods used to assess risk of bias in the included studies, including details of the tool(s) used, how many reviewers assessed each study and whether they worked independently, and if applicable, details of automation tools used in the process. | 9-10 |
| Effect measures | 12 | Specify for each outcome the effect measure(s) (e.g., risk ratio, mean difference) used in the synthesis or presentation of results. | 10 |
| Synthesis methods | 13a | Describe the processes used to decide which studies were eligible for each synthesis (e.g., tabulating the study intervention characteristics and comparing against the planned groups for each synthesis (item #5)). | 9 |
|  | 13b | Describe any methods required to prepare the data for presentation or synthesis, such as handling of missing summary statistics, or data conversions. | NA |
|  | 13c | Describe any methods used to tabulate or visually display results of individual studies and syntheses. | NA |
|  | 13d | Describe any methods used to synthesize results and provide a rationale for the choice(s). If meta-analysis was performed, describe the model(s), method(s) to identify the presence and extent of statistical heterogeneity, and software package(s) used. | 10 |
|  | 13e | Describe any methods used to explore possible causes of heterogeneity among study results (e.g., subgroup analysis, meta-regression). | NA |
|  | 13f | Describe any sensitivity analyses conducted to assess robustness of the synthesized results. | NA |
| Reporting bias assessment | 14 | Describe any methods used to assess risk of bias due to missing results in a synthesis (arising from reporting biases). | NA |
| Certainty assessment | 15 | Describe any methods used to assess certainty (or confidence) in the body of evidence for an outcome. | NA |
| **RESULTS** | | |  |
| Study selection | 16a | Describe the results of the search and selection process, from the number of records identified in the search to the number of studies included in the review, ideally using a flow diagram. | Supplementary Fig. 1 |
|  | 16b | Cite studies that might appear to meet the inclusion criteria, but which were excluded, and explain why they were excluded. | Supplementary Table 3 |
| Study characteristics | 17 | Cite each included study and present its characteristics. | 11, Table 1 |
| Risk of bias | 18 | Present assessments of risk of bias for each included study. | Supplementary Table 4, Supplementary Fig. 5 |
| Results of individual studies | 19 | For all outcomes, present, for each study: (a) summary statistics for each group (where appropriate) and (b) an effect estimates and its precision (e.g., confidence/credible interval), ideally using structured tables or plots. | 11, Fig. 1-2, Supplementary Fig. 4 |
| Results of syntheses | 20a | For each synthesis, briefly summarise the characteristics and risk of bias among contributing studies. | NA |
|  | 20b | Present results of all statistical syntheses conducted. If meta-analysis was done, present for each the summary estimate and its precision (e.g., confidence/credible interval) and measures of statistical heterogeneity. If comparing groups, describe the direction of the effect. | 11-12,  Fig. 1-2, Table 2, Supplementary Fig. 3, 4, 6 |
|  | 20c | Present results of all investigations of possible causes of heterogeneity among study results. | Supplementary Fig. 6 |
|  | 20d | Present results of all sensitivity analyses conducted to assess the robustness of the synthesized results. | Supplementary Fig. 2 |
| Reporting biases | 21 | Present assessments of risk of bias due to missing results (arising from reporting biases) for each synthesis assessed. | NA |
| Certainty of evidence | 22 | Present assessments of certainty (or confidence) in the body of evidence for each outcome assessed. | NA |
| **DISCUSSION** | | |  |
| Discussion | 23a | Provide a general interpretation of the results in the context of other evidence. | 13-14 |
|  | 23b | Discuss any limitations of the evidence included in the review. | 14-15 |
|  | 23c | Discuss any limitations of the review processes used. | 15 |
|  | 23d | Discuss implications of the results for practice, policy, and future research. | 16-17 |
| **OTHER INFORMATION** | | |  |
| Registration and protocol | 24a | Provide registration information for the review, including register name and registration number, or state that the review was not registered. | 8 |
|  | 24b | Indicate where the review protocol can be accessed, or state that a protocol was not prepared. | 8 |
|  | 24c | Describe and eqxplain any amendments to information provided at registration or in the protocol. | NA |
| Support | 25 | Describe sources of financial or non-financial support for the review, and the role of the funders or sponsors in the review. | 18 |
| Competing interests | 26 | Declare any competing interests of review authors. | 18-19 |
| Availability of data, code and other materials | 27 | Report which of the following are publicly available and where they can be found: template data collection forms; data extracted from included studies; data used for all analyses; analytic code; any other materials used in the review. | Table 1 |

**Supplementary Table 2. Detailed search strategy**

| **Database** | **Keyword** | **Filter** | **Date** | **Results** |
| --- | --- | --- | --- | --- |
| PubMed | (SEP-363856 OR SEP-856 OR Ulotaront) AND (psychosis OR psychotic disorder OR schizophreni* OR schizoaffective disorder OR delusional disorder) | Not applied | January 22, 2025 | 65 |
| Embase | (SEP-363856 OR SEP-856 OR Ulotaront) AND (psychosis OR psychotic disorder OR schizophreni* OR schizoaffective disorder OR delusional disorder) | Not applied | January 22, 2025 | 144 |
| Cochrane CENTRAL | (SEP-363856 OR SEP-856 OR Ulotaront) AND (psychosis OR psychotic disorder OR schizophreni* OR schizoaffective disorder OR delusional disorder) | Not applied | January 22, 2025 | 51 |
| ClinicalTrials.gov | (SEP-363856 OR SEP-856 OR Ulotaront) AND (psychosis OR psychotic disorder OR schizophreni* OR schizoaffective disorder OR delusional disorder) | Not applied | January 22, 2025 | 23 |

We also post-hoc conducted a manual search using Google to gather additional information about included randomized controlled trials, aiming to obtain the most up-to-date data, such as reports from the American College of Neuropsychopharmacology's website (<https://www.cnsscientificposters.com/acnp-2023>).

**Supplementary Table 3. Excluded studies and reasons**

| Reasons | Reference |
| --- | --- |
| Non randomized controlled trial | Correll, C. U., et al. (2021). "Safety and effectiveness of ulotaront (SEP-363856) in schizophrenia: results of a 6-month, open-label extension study." NPJ Schizophr **7**(1): 63. |
|  | Milanovic, S., et al. (2020). "Measures of cognition and social functioning in schizophrenia patients receiving SEP-363856." Schizophrenia Bulletin **46**: S215-S216.  Goff, D. C. (2020). "Promising evidence of antipsychotic efficacy without dopamine d2-receptor binding." New England Journal of Medicine 382(16): 1555-1556. |
| No outcome of interest | jRct (2021). "A Clinical Trial to Evaluate the Efficacy and Safety of SEP-363856 in Acutely Psychotic People With Schizophrenia, Followed by an Open-label Extension Phase." https://trialsearch.who.int/Trial2.aspx?TrialID=JPRN-jRCT2071210003. |
| Duplication report | Nct (2021). "A Clinical Trial to Evaluate the Efficacy and Safety of SEP-363856 in Acutely Psychotic People With Schizophrenia, Followed by an Open-label Extension Phase." <https://clinicaltrials.gov/ct2/show/NCT04825860>.  Diener, H. C. (2020). "Schizophrenia: Assessment of SEP-363856, a TAAR1- and 5-HT1A receptor antagonist in acute therapy." Psychopharmakotherapie **27**(3): 166-167.  (2021). A Randomized, Double-blind, Parallel-group, Placebo Controlled, Fixed-dose, Multicenter Study to Evaluate the Efficacy and Safety of SEP 363856 in Acutely Psychotic Patients With Schizophrenia, Followed by an Open-label Extension Phase. |

**Supplementary Table 4. Detailed quality assessment of included studies using Cochrane risk of bias 2 tool**

| First Author | Year | Randomization  process | Intervention  adherence | Missing  outcome data | Outcome  measurement | Selective  reporting | Overall  RoB |
| --- | --- | --- | --- | --- | --- | --- | --- |
| Koblan 2020^1^ | 2020 | L | L | L | L | L | L |
| DIAMOND 1^#^  (NCT04072354)^2^ | 2024 | L | L | L | L | L | L |
| DIAMOND 2^#^  (NCT04092686)^3^ | 2024 | L | L | L | L | L | L |

H, high risk of bias; L, low risk of bias; RoB, risk of bias; S, some concerns.

^#^ A poster presented at the American College of Neuropsychopharmacology provides data from the DIAMOND 1 and DIAMOND 2 studies. The poster can be accessed online via the following link: https://cnsscientificposters.ipostersessions.com/Default.aspx?s=30-4A-E4-FB-51-B0-CE-51-94-ED-E5-96-B7-42-A4-D0

**Supplementary Figure 1.** **Flowchart of study selection**

**
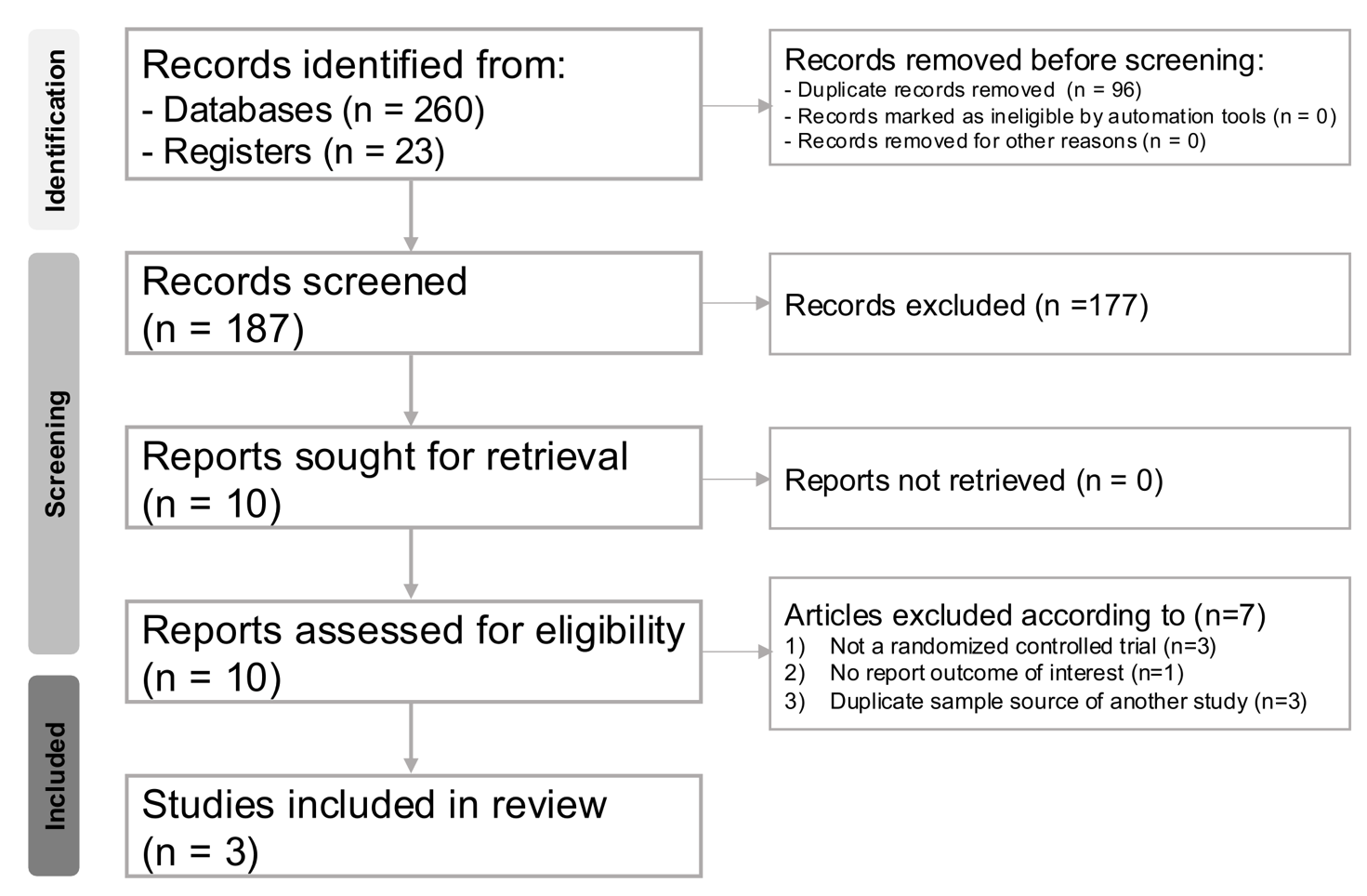
**

**Supplementary Figure 2. Leave-one-out analysis of the dose-response relationship between ulotaront dose and PANSS total score**


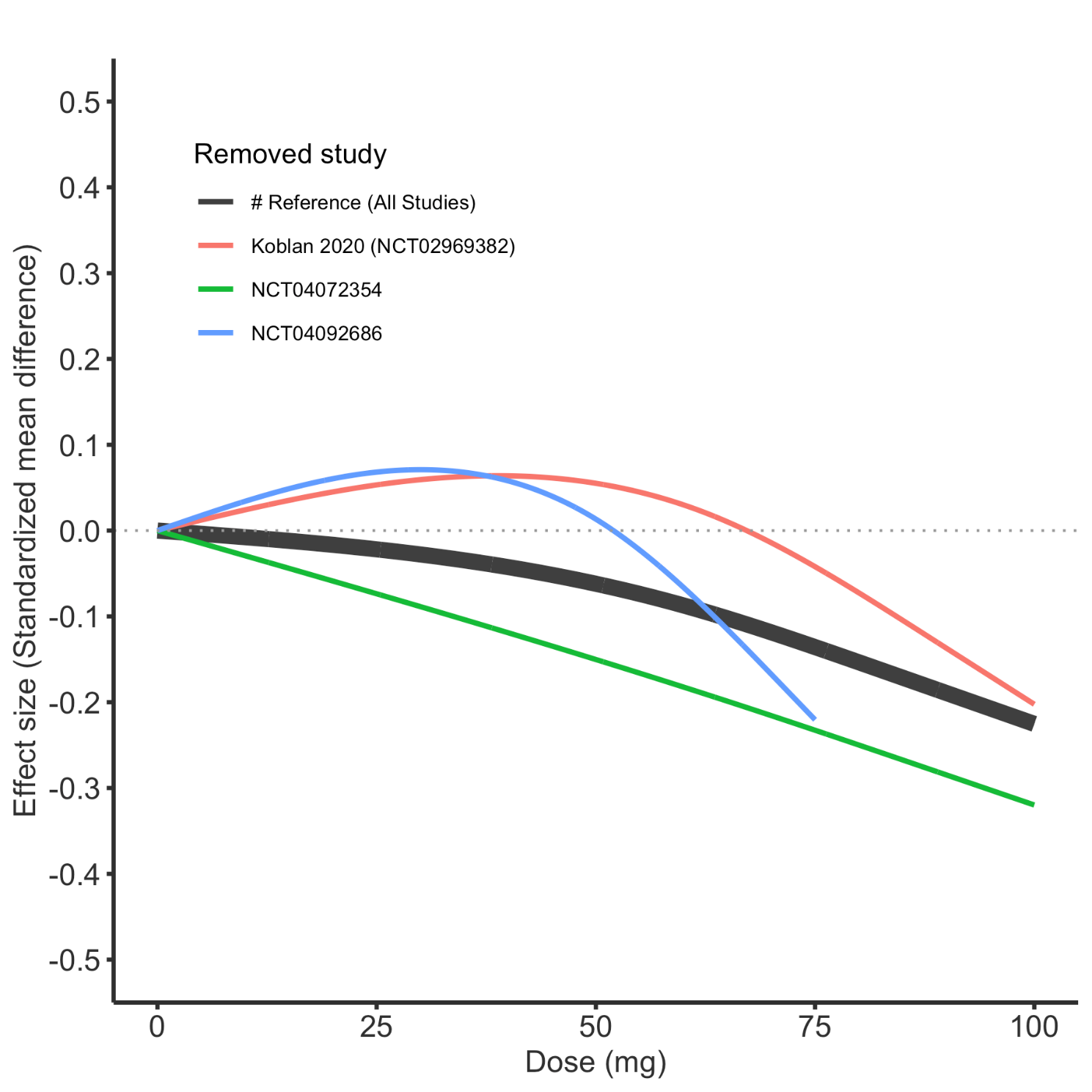


**Supplementary Figure 3. Time-effect relationship of PANSS total score**

**
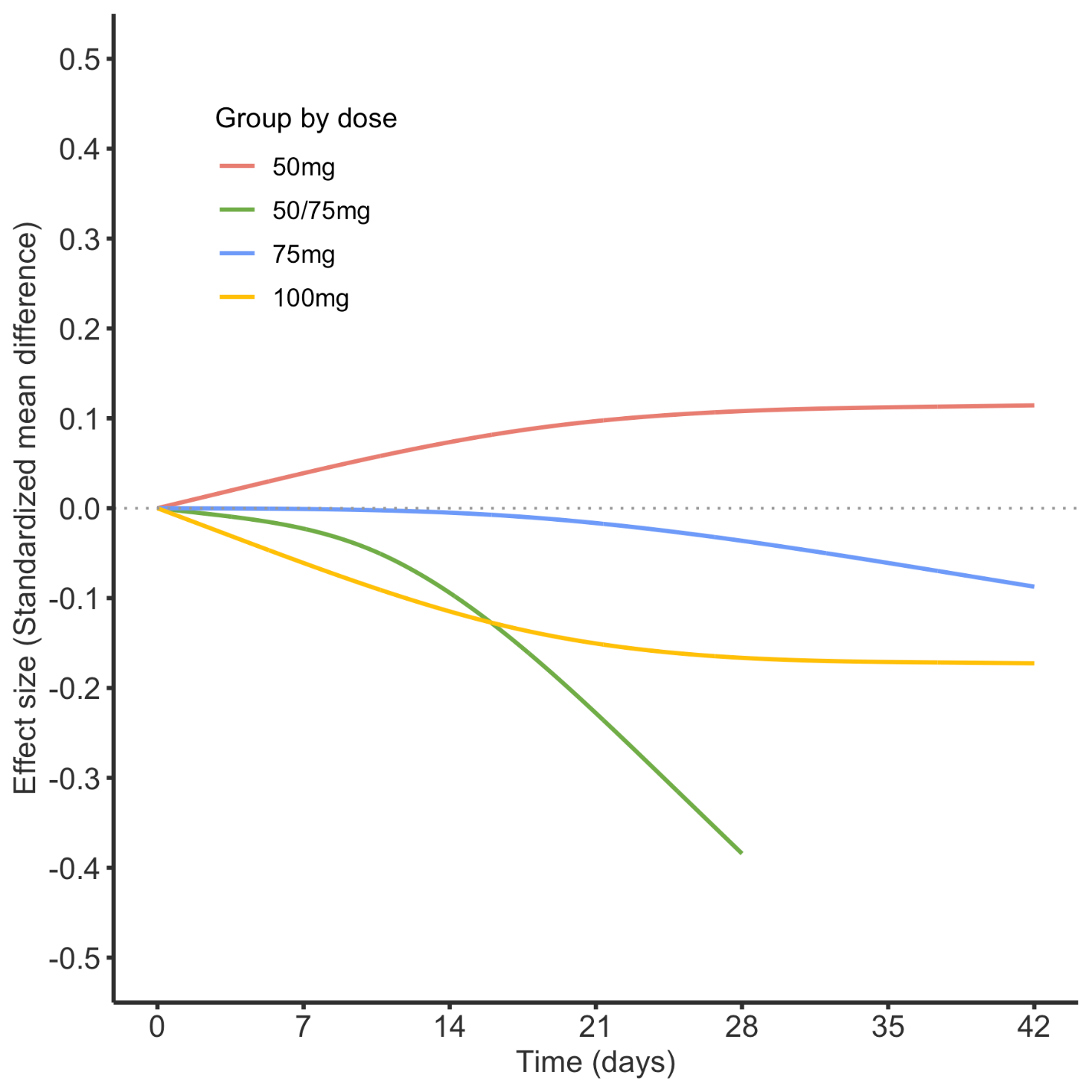
**

**Supplementary Figure 4. Dose-response relationships between ulotaront doses and specific adverse events**

1. **Extrapyramidal symptoms**

**
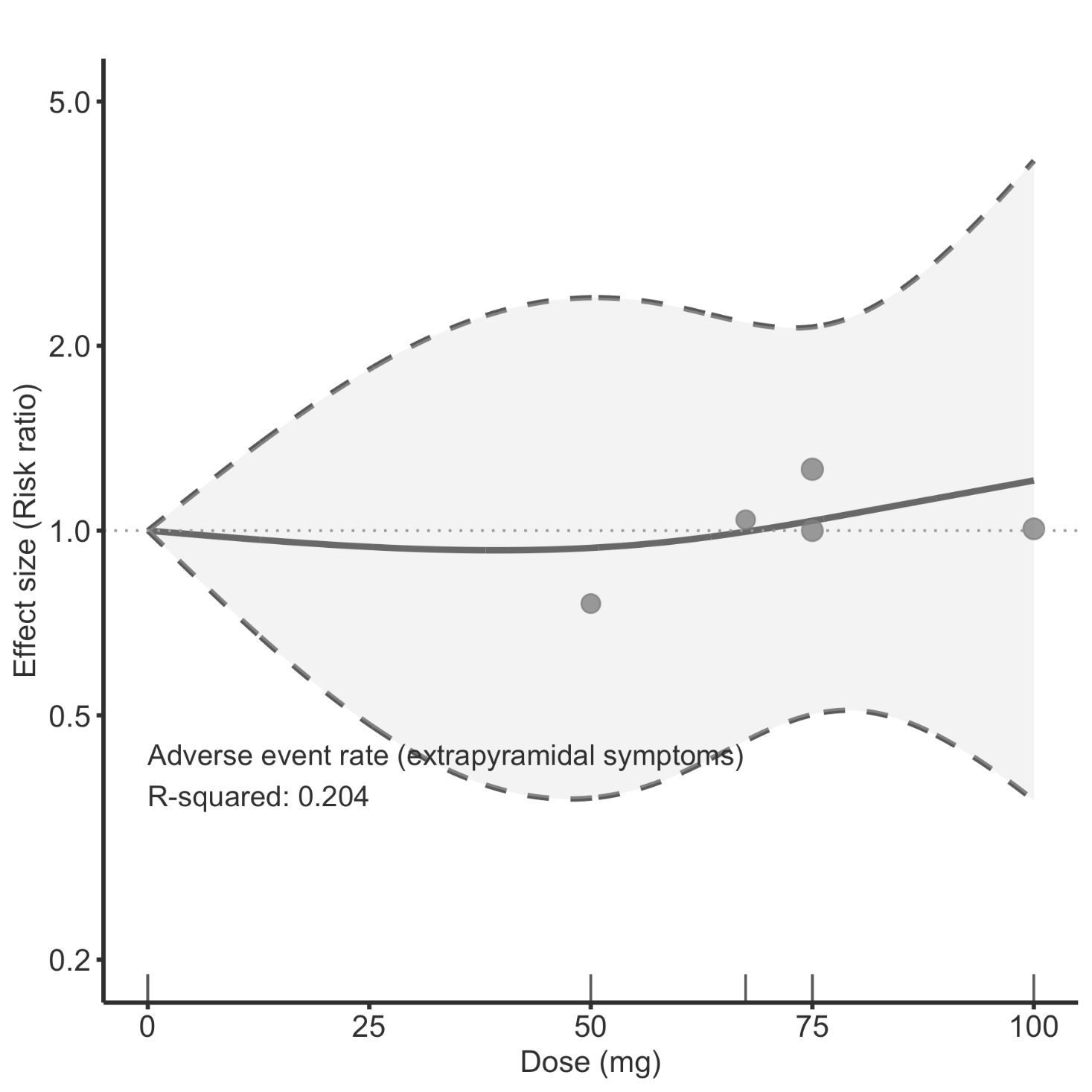
**

1. **Insomnia**

**
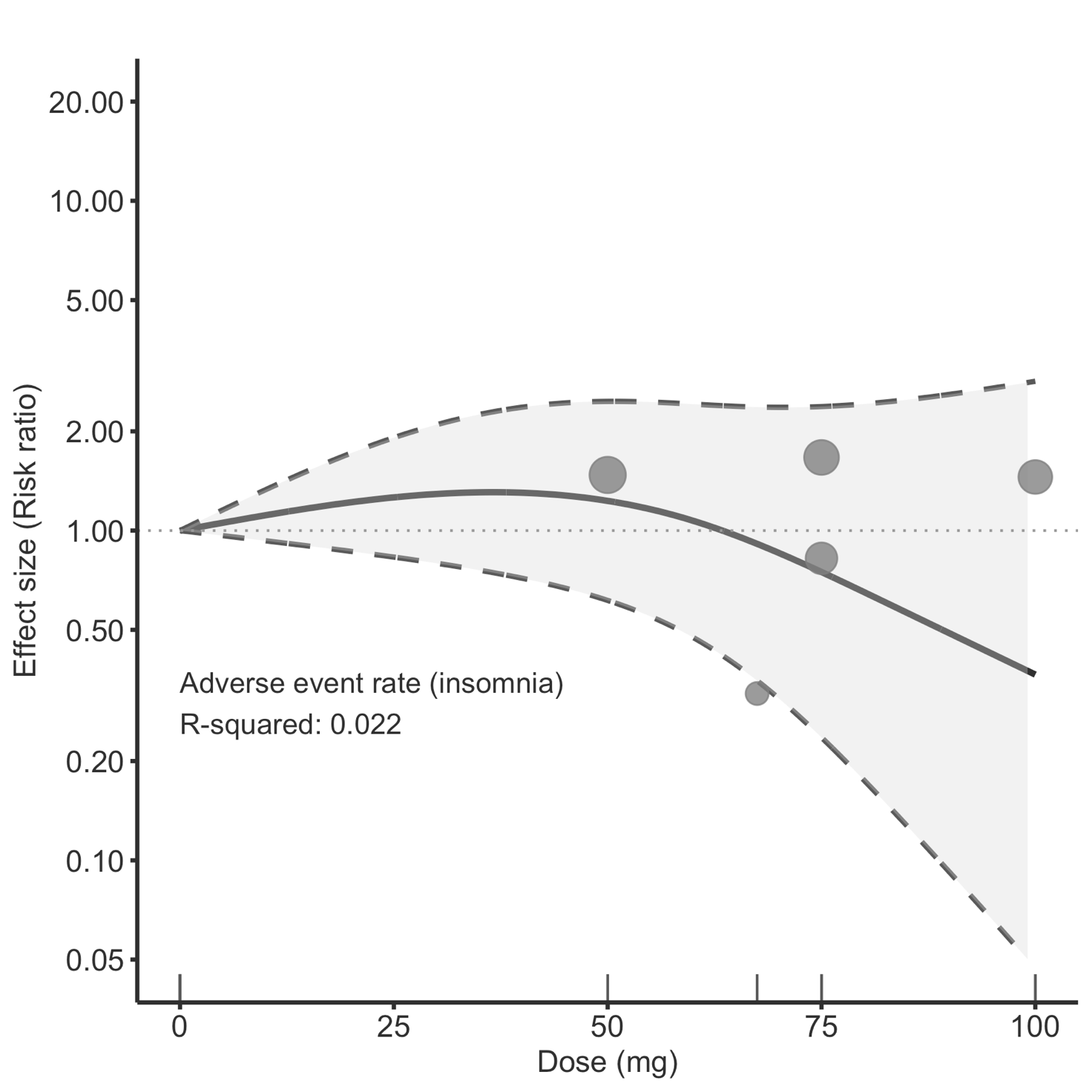
**

1. **Headache**

**
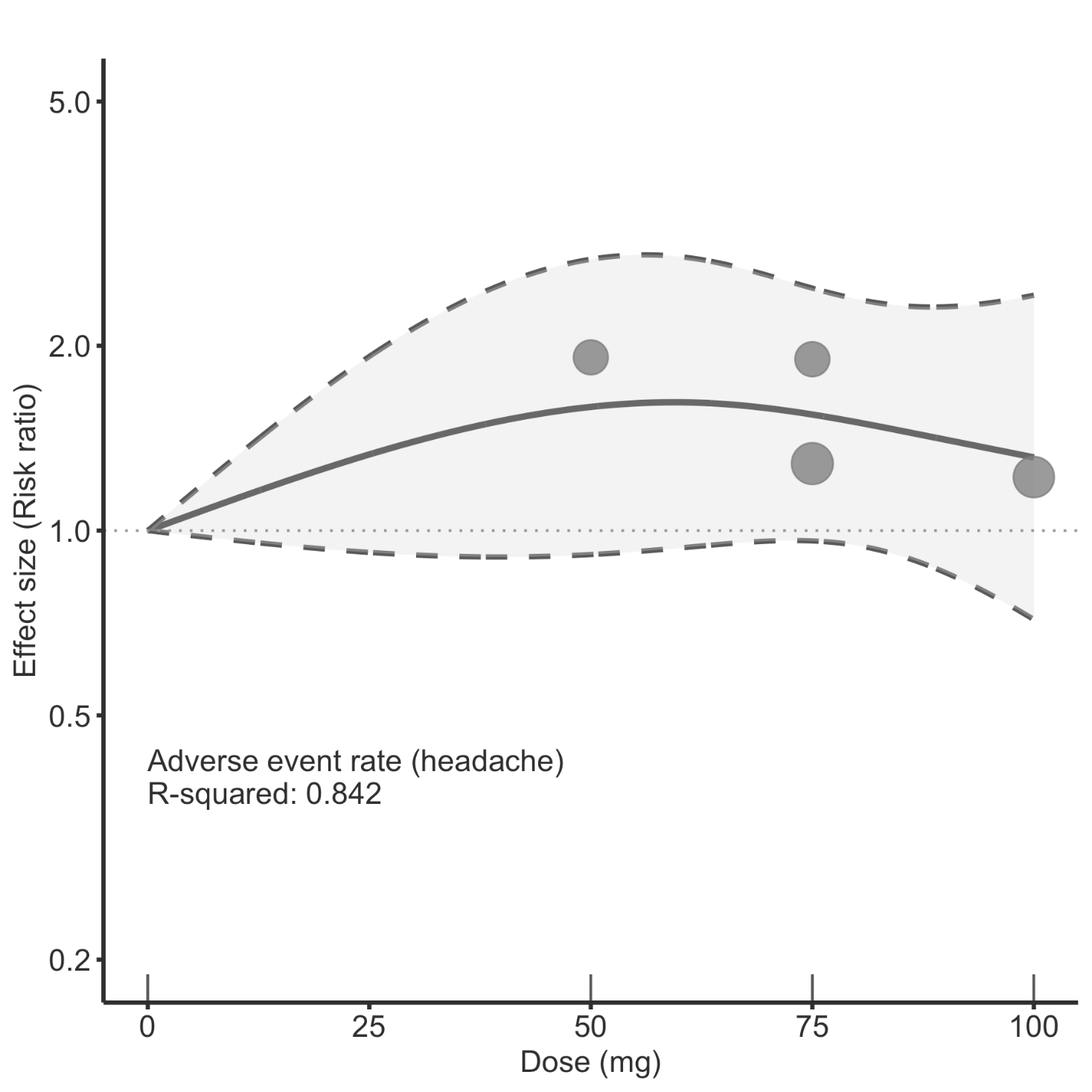
**

1. **Nausea**

**
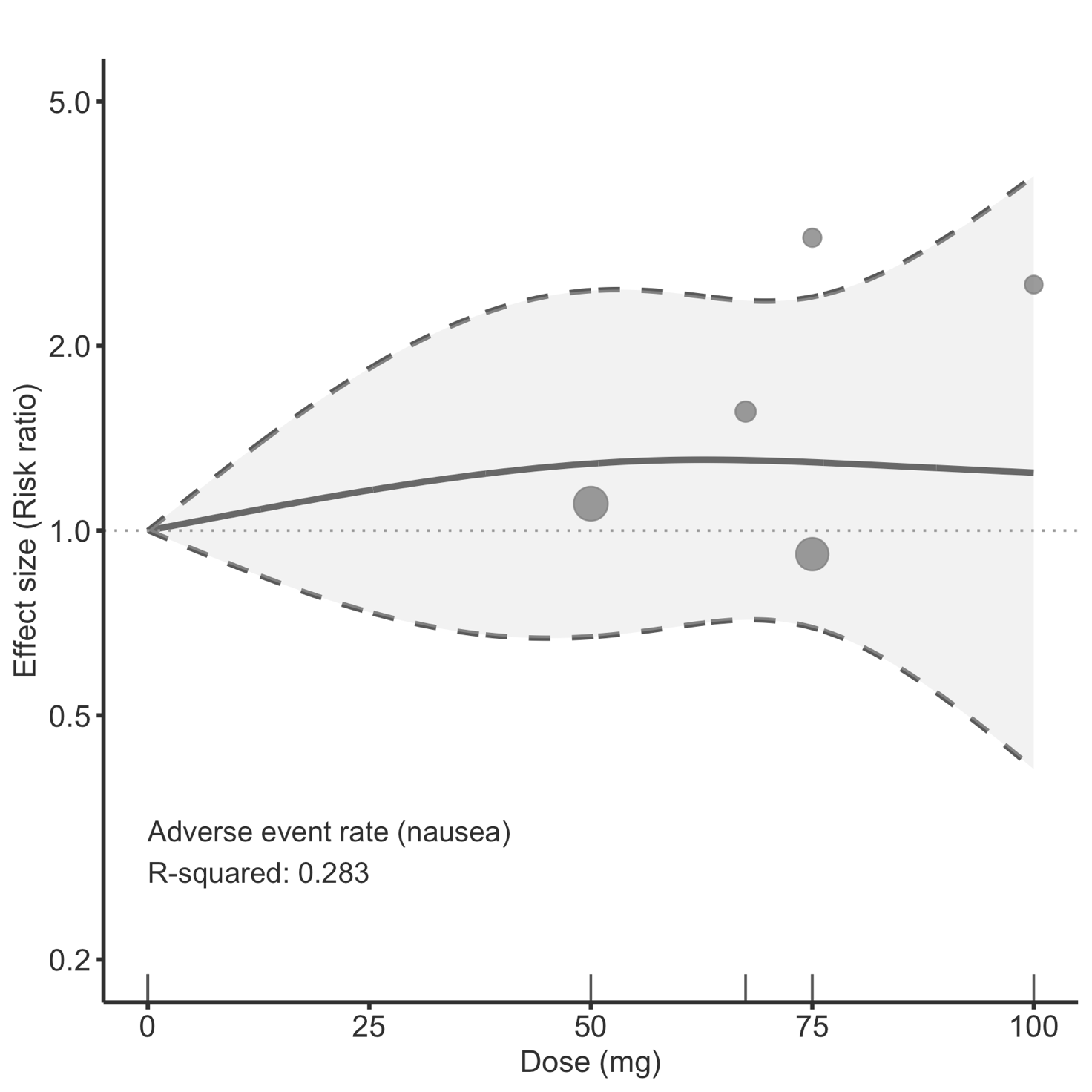
**

1. **Agitation**


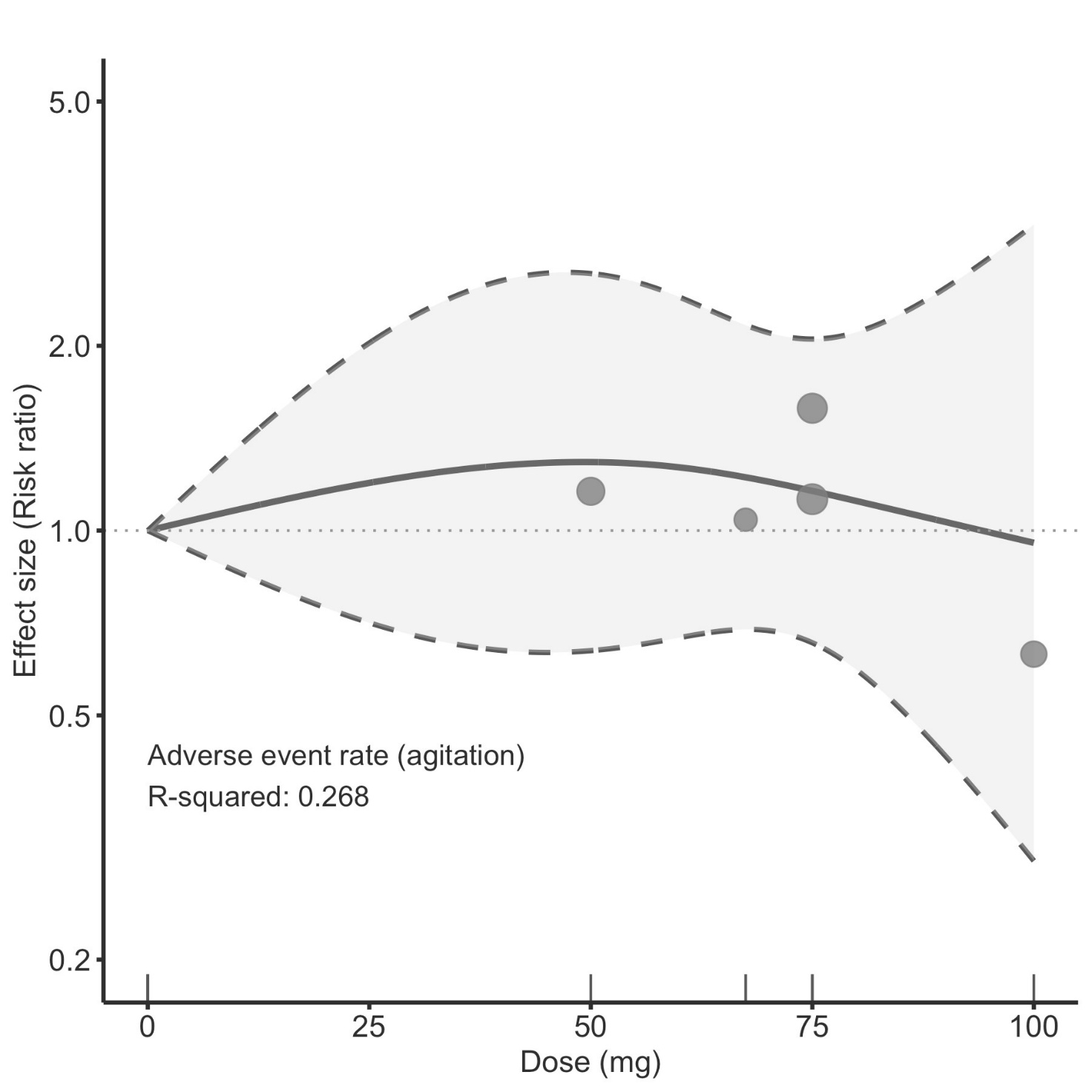


1. **Schizophrenia**


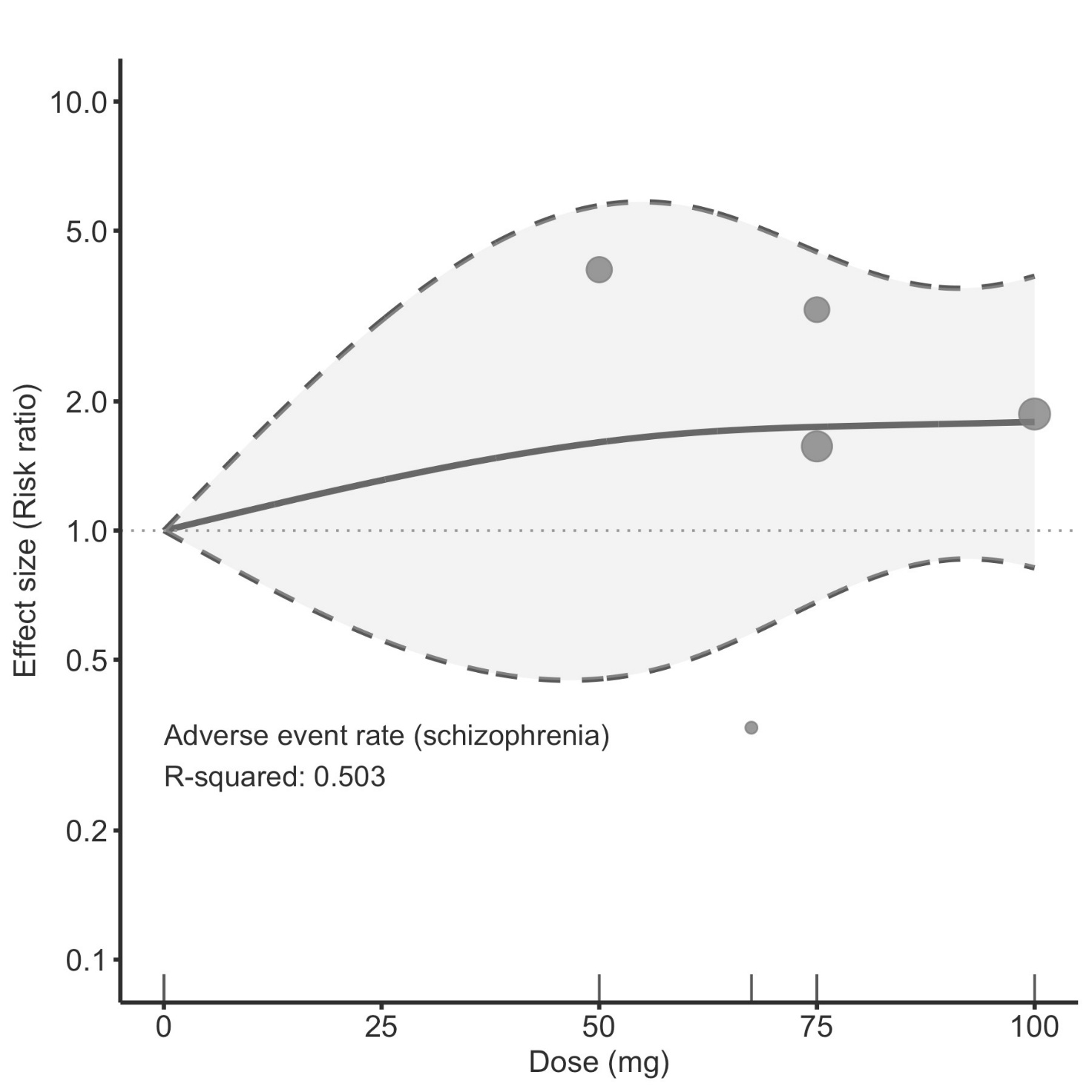


1. **Anxiety**


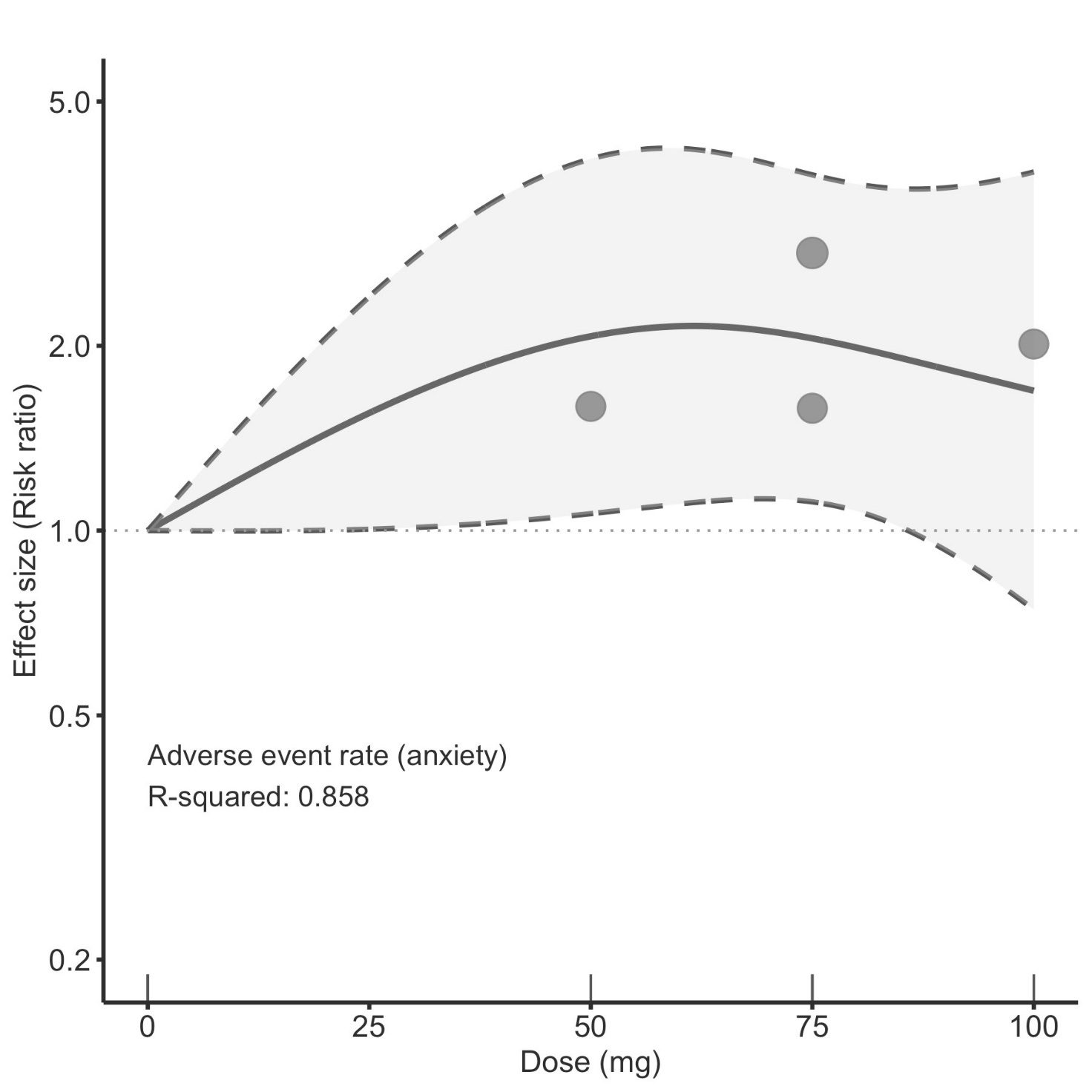


**Supplementary Figure 5. Summary of quality assessment of included studies using Cochrane risk of bias 2 tool**

**Supplementary Figure 6. Variation partition coefficients of dose-response meta-analysis**

1. **PANSS total score**

**
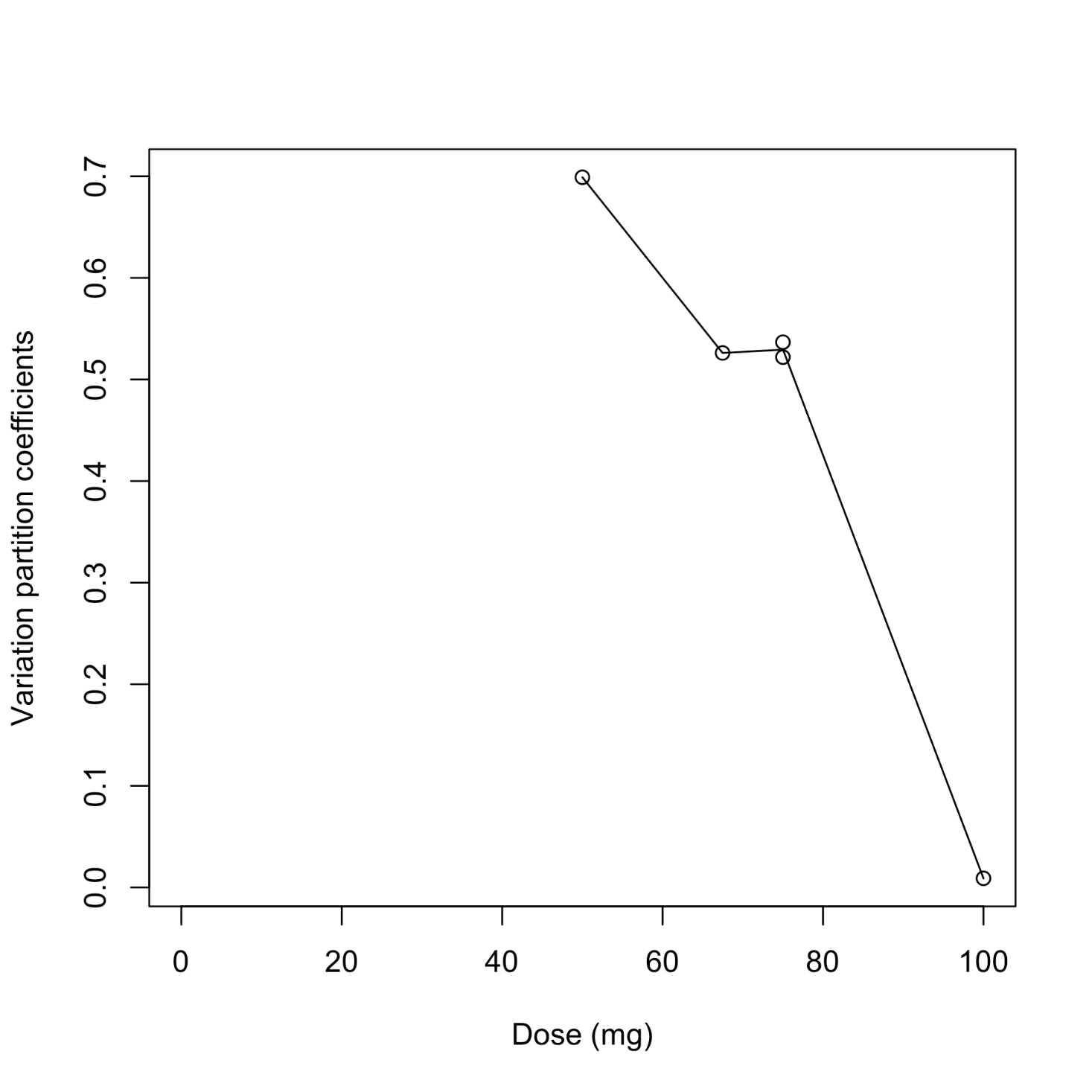
**

1. **Dropout**

**
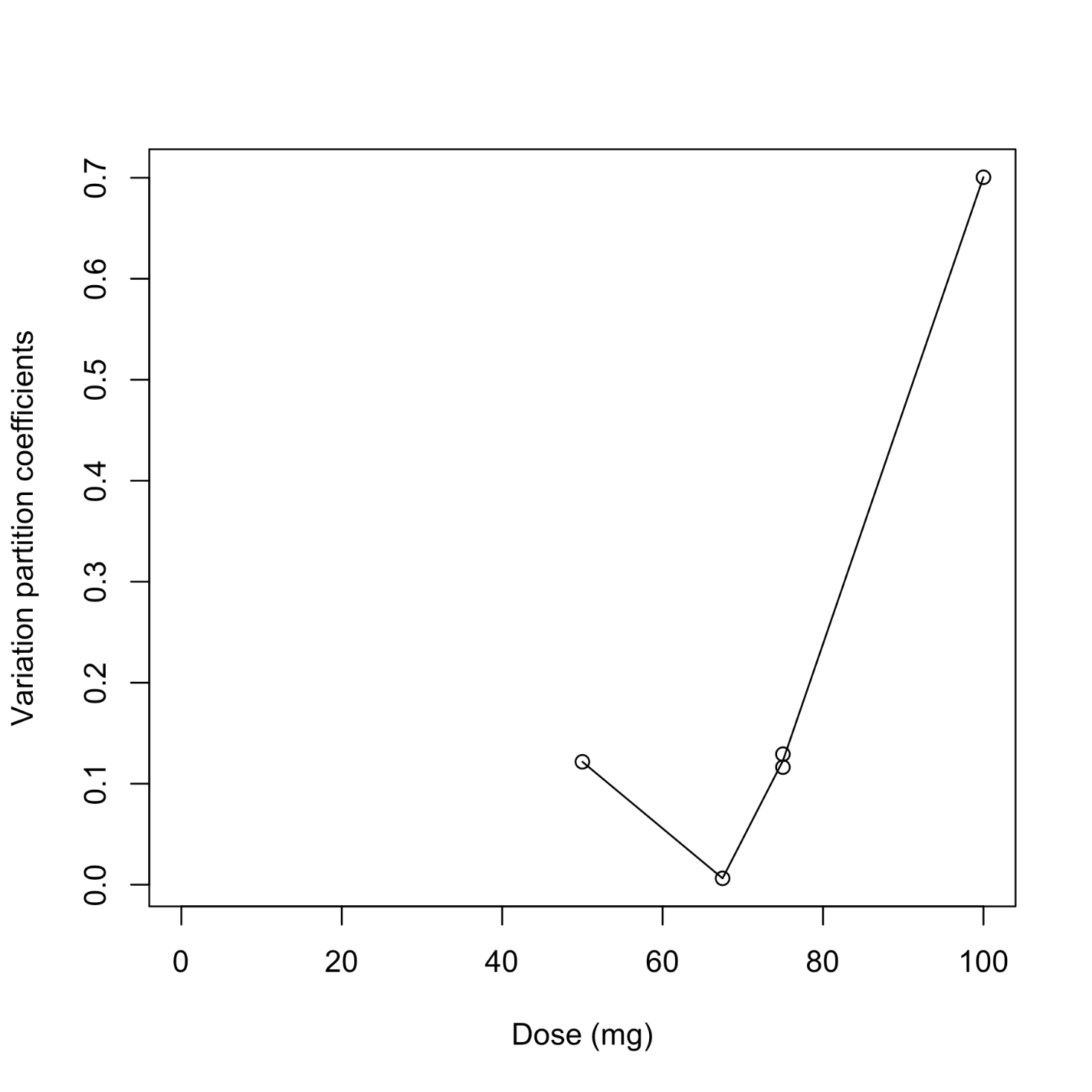
**

**References**

1. Koblan KS, Kent J, Hopkins SC, et al. A Non-D2-Receptor-Binding Drug for the Treatment of Schizophrenia. *N Engl J Med*. Apr 16 2020;382(16):1497-1506. doi:10.1056/NEJMoa1911772

2. NCT04072354

2019-000470-36

A Randomised, Double-blind, Parallel-group, Placebo-controlled, Fixeddose, Multicenter Study to Evaluate the Efficacy and Safety of SEP363856 in Acutely Psychotic Subjects with Schizophrenia. *EU Clinical Trials Register*. 2023;

3. NCT04092686

2019-000697-37

A Randomized, Double-blind, Parallel-group, Placebo-controlled, Fixed

dose, Multicenter Study to Evaluate the Efficacy and Safety of SEP

363856 in Acutely Psychotic Subjects with Schizophrenia. *EU Clinical Trials Register*. 2023;
